# (1-Methyl-1*H*-imidazol-3-ium-2-yl)(phen­yl)phosphinate monohydrate

**DOI:** 10.1107/S1600536812028255

**Published:** 2012-07-04

**Authors:** Yong-Ming Sun, Meng Yang, Chang-Qiu Zhao

**Affiliations:** a College of Chemistry and Chemical Engineering, Liaocheng University, Shandong 252059, People’s Republic of China

## Abstract

The title compound, C_10_H_11_N_2_O_2_P·H_2_O, contains a tetra­coordinate penta­valent P atom. The phosphinate group plays a predominant role in the cohesion of the crystal structure by forming chains along the *b* axis *via* inter­molecular C—H⋯O hydrogen bonds. These chains are connected by O—H⋯O and N—H⋯O hydrogen bonding involving the lattice water.

## Related literature

For background infomation on phospho­rylated imidazoles, see: Andrej *et al.* (1999 ▶); Matevosyan & Zavlin (1990 ▶); Grotjahn (2010 ▶). For the structures of related imidazolyl phosphinic acids and the function of phospho­rylated imidazoles, see: Kunz & Frank (2010 ▶).
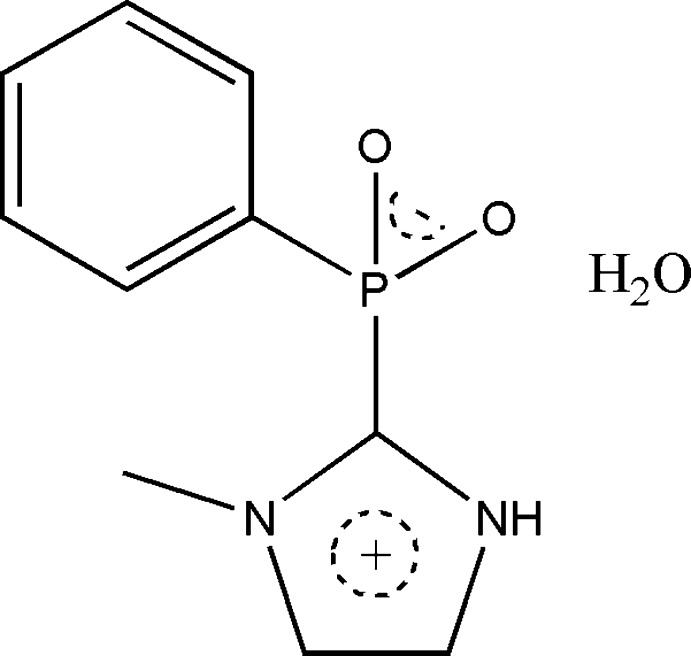


## Experimental

### 

#### Crystal data

C_10_H_11_N_2_O_2_P·H_2_O
*M* *_r_* = 240.19
Monoclinic, 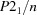

*a* = 6.7946 (5) Å
*b* = 24.753 (2) Å
*c* = 7.5277 (7) Å
β = 114.433 (1)°
*V* = 1152.70 (17) Å^3^
*Z* = 4
Mo *K*α radiation
μ = 0.23 mm^−1^
*T* = 298 K
0.40 × 0.31 × 0.14 mm

#### Data collection

Bruker SMART CCD area-detector diffractometer
Absorption correction: multi-scan (*SADABS*; Sheldrick, 1996 ▶) *T*_min_ = 0.913, *T*_max_ = 0.968
6904 measured reflections
2597 independent reflections
1489 reflections with *I* > 2σ(*I*)
*R*_int_ = 0.048

#### Refinement

*R*[*F* ^2^ > 2σ(*F* ^2^)] = 0.054
*wR*(*F* ^2^) = 0.154
*S* = 1.02
2597 reflections
146 parameters
H-atom parameters constrained
Δρ_max_ = 0.32 e Å^−3^
Δρ_min_ = −0.32 e Å^−3^

### 

Data collection: *SMART* (Siemens, 1996 ▶); cell refinement: *SAINT* (Siemens, 1996 ▶); data reduction: *SAINT*; program(s) used to solve structure: *SHELXS97* (Sheldrick, 2008 ▶); program(s) used to refine structure: *SHELXL97* (Sheldrick, 2008 ▶); molecular graphics: *SHELXTL* (Sheldrick, 2008 ▶); software used to prepare material for publication: *SHELXTL*.

## Supplementary Material

Crystal structure: contains datablock(s) I, global. DOI: 10.1107/S1600536812028255/mw2070sup1.cif

Structure factors: contains datablock(s) I. DOI: 10.1107/S1600536812028255/mw2070Isup2.hkl

Supplementary material file. DOI: 10.1107/S1600536812028255/mw2070Isup3.cml

Additional supplementary materials:  crystallographic information; 3D view; checkCIF report

## Figures and Tables

**Table 1:** Hydrogen-bond geometry (Å, °)

*D*—H⋯*A*	*D*—H	H⋯*A*	*D*⋯*A*	*D*—H⋯*A*
O3—H3*A*⋯O1^i^	0.85	1.86	2.709 (3)	174
C10—H10*B*⋯O1^ii^	0.96	2.51	3.268 (4)	136
N2—H20⋯O3^iii^	0.87	1.82	2.665 (3)	162

Symmetry codes: (i) 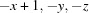; (ii) 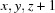; (iii) 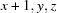.

**Fig. 1.**
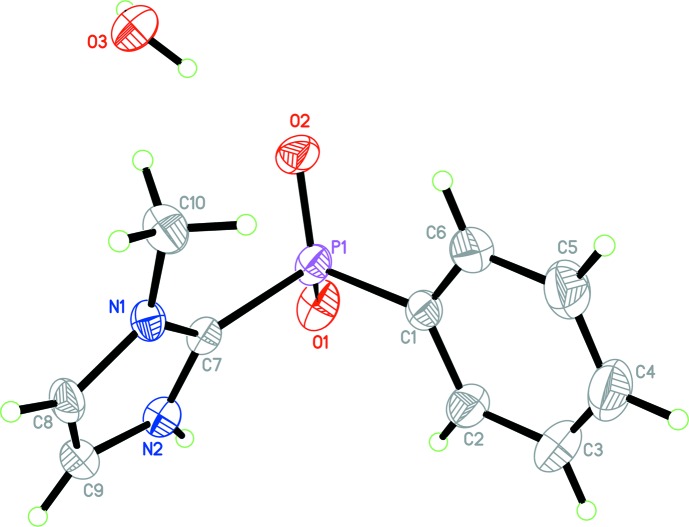
The molecular structure of the title compound. Displacement ellipsoids are drawn at the 50% probability level.

**Fig. 2.**
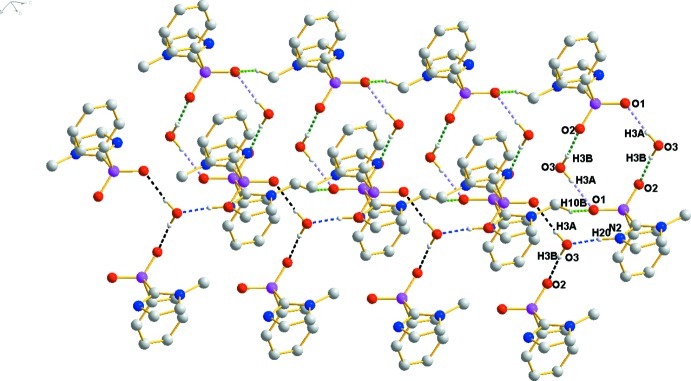
The two–dimensional plane, linked by C—H···O, N—H···O and O—H···O interactions.

## supplementary crystallographic information

### Comment

Phosphorylated imidazoles have attracted the attention of chemists and biochemists in the past decades because they are promising as synthons, pesticides, and drugs (Matevosyan & Zavlin, 1990). Pyridylphosphanes are well-established P—N ligands in transition metal chemistry (Grotjahn, 2010) while phosphinic acids have found use in the construction of coordination polymers for a wide range of applications (Kunz & Frank, 2010). The title compound, C_10_H_13_N_2_O_3_P, contains a tetracoordinate pentavalent P atom and the phosphinic function plays a predominant role in the cohesion of the crystal structure, both by forming chains along the *b* axis via weak intermolecular C—H···O hydrogen bonds and by connecting these chains by O—H···O and N—H···O hydrogen bonding with the lattice water molecules. The O—H···O and C—H···O interactions form 12-membered hydrogen bonded rings that are located on centers of inversion (Kunz & Frank, 2010) while the O—H···O and N—H···O interaction form 14-membered hydrogen bonded rings (Fig. 2).

### Experimental

Triethylamine (14.74 mmol) was added dropwise at 0°C to dichlorophenylphosphine (7.37 mmol) dissolved in dichloromethane (10 ml) and the mixture was stirred for 10 min. Methylimidazole (14.74 mmol) was added and the reaction mixture was warmed to room temperature and stirred for 10 h. After removal of the solvent, ethanol (30 ml) and sodium hydroxide (14.74 mmol) were added and the mixture stirred for 3 h. The solvent was removed *in vacuo*, dichloromethane (10 ml) was added, the precipitate was filtered off and the solvent removed *in vacuo* to give the crude product (2,2'-(phenylphosphinediyl)bis(1-methyl-1*H*-imidazole)). Butyl ether was used to recrystallize. Single crystals of the title compound suitable for *X*–ray diffraction were obtained by slow evaporation of a methanol solution of the product.

### Refinement

All H atoms attached to C, N and O atoms were fixed geometrically and treated as riding with C—H = 0.93–0.96 Å, O—H = 0.85 Å, N—H = 0.87 Å, with *U*_iso_(H) = 1.5 *U*_eq_(methyl) and *U*_iso_(H) = 1.2 *U*_eq_(C) for all other H atoms.

### Crystal data

**Table:**

C_10_H_11_N_2_O_2_P·H_2_O	*F*(000) = 504
*M**_r_* = 240.19	*D*_x_ = 1.384 Mg m^−^^3^
Monoclinic, *P*2_1_/*n*	Mo *K*α radiation, λ = 0.71073 Å
Hall symbol: -P 2yn	Cell parameters from 1313 reflections
*a* = 6.7946 (5) Å	θ = 2.5–23.3°
*b* = 24.753 (2) Å	µ = 0.23 mm^−^^1^
*c* = 7.5277 (7) Å	*T* = 298 K
β = 114.433 (1)°	Block, colourless
*V* = 1152.70 (17) Å^3^	0.40 × 0.31 × 0.14 mm
*Z* = 4	

### Data collection

**Table:**

Bruker SMART CCD area-detector diffractometer	2597 independent reflections
Radiation source: fine-focus sealed tube	1489 reflections with *I* > 2σ(*I*)
Graphite monochromator	*R*_int_ = 0.048
φ and ω scans	θ_max_ = 27.5°, θ_min_ = 3.1°
Absorption correction: multi-scan (*SADABS*; Sheldrick, 1996)	*h* = −8→8
*T*_min_ = 0.913, *T*_max_ = 0.968	*k* = −29→32
6904 measured reflections	*l* = −9→7

### Refinement

**Table:**

Refinement on *F*^2^	Primary atom site location: structure-invariant direct methods
Least-squares matrix: full	Secondary atom site location: difference Fourier map
*R*[*F*^2^ > 2σ(*F*^2^)] = 0.054	Hydrogen site location: inferred from neighbouring sites
*wR*(*F*^2^) = 0.154	H-atom parameters constrained
*S* = 1.02	*w* = 1/[σ^2^(*F*_o_^2^) + (0.0721*P*)^2^ + 0.0591*P*] where *P* = (*F*_o_^2^ + 2*F*_c_^2^)/3
2597 reflections	(Δ/σ)_max_ = 0.001
146 parameters	Δρ_max_ = 0.32 e Å^−^^3^
0 restraints	Δρ_min_ = −0.32 e Å^−^^3^

### Special details

**Table:**

Geometry. All esds (except the esd in the dihedral angle between two l.s. planes) are estimated using the full covariance matrix. The cell esds are taken into account individually in the estimation of esds in distances, angles and torsion angles; correlations between esds in cell parameters are only used when they are defined by crystal symmetry. An approximate (isotropic) treatment of cell esds is used for estimating esds involving l.s. planes.
Refinement. Refinement of *F*^2^ against ALL reflections. The weighted *R*-factor *wR* and goodness of fit *S* are based on *F*^2^, conventional *R*-factors *R* are based on *F*, with *F* set to zero for negative *F*^2^. The threshold expression of *F*^2^ > σ(*F*^2^) is used only for calculating *R*-factors(gt) *etc*. and is not relevant to the choice of reflections for refinement. *R*-factors based on *F*^2^ are statistically about twice as large as those based on *F*, and *R*- factors based on ALL data will be even larger.

### Fractional atomic coordinates and isotropic or equivalent isotropic displacement parameters (Å^2^)

**Table:**

	*x*	*y*	*z*	*U*_iso_*/*U*_eq_	
P1	0.73237 (13)	0.10122 (3)	0.11830 (10)	0.0428 (3)	
O1	0.8280 (4)	0.07348 (9)	−0.0024 (3)	0.0608 (7)	
O2	0.5092 (3)	0.09076 (9)	0.0956 (3)	0.0578 (6)	
N1	0.8906 (4)	0.09159 (8)	0.5352 (3)	0.0346 (5)	
N2	1.1068 (4)	0.06002 (9)	0.4191 (3)	0.0403 (6)	
H20	1.1542	0.0490	0.3337	0.048*	
C1	0.7732 (5)	0.17278 (12)	0.1149 (4)	0.0428 (7)	
C2	0.9522 (5)	0.19315 (13)	0.0927 (5)	0.0562 (9)	
H2	1.0506	0.1695	0.0775	0.067*	
C3	0.9849 (7)	0.24800 (15)	0.0931 (6)	0.0784 (12)	
H3	1.1050	0.2613	0.0778	0.094*	
C4	0.8414 (8)	0.28317 (15)	0.1158 (6)	0.0796 (12)	
H4	0.8644	0.3202	0.1153	0.096*	
C5	0.6659 (7)	0.26430 (14)	0.1391 (6)	0.0727 (11)	
H5	0.5698	0.2884	0.1557	0.087*	
C6	0.6298 (5)	0.20933 (14)	0.1381 (5)	0.0580 (9)	
H6	0.5086	0.1966	0.1529	0.070*	
C7	0.9148 (4)	0.08320 (10)	0.3684 (4)	0.0331 (6)	
C8	1.0715 (5)	0.07368 (11)	0.6899 (4)	0.0435 (7)	
H8	1.0965	0.0750	0.8210	0.052*	
C9	1.2055 (5)	0.05385 (12)	0.6160 (4)	0.0459 (7)	
H9	1.3409	0.0387	0.6864	0.055*	
C10	0.7023 (5)	0.11496 (13)	0.5516 (4)	0.0502 (8)	
H10A	0.6580	0.1468	0.4721	0.075*	
H10B	0.7382	0.1243	0.6852	0.075*	
H10C	0.5864	0.0892	0.5083	0.075*	
O3	0.3180 (3)	0.01614 (8)	0.2251 (3)	0.0536 (6)	
H3A	0.2753	−0.0110	0.1494	0.080*	
H3B	0.3774	0.0385	0.1767	0.080*	

### Atomic displacement parameters (Å^2^)

**Table:**

	*U*^11^	*U*^22^	*U*^33^	*U*^12^	*U*^13^	*U*^23^
P1	0.0533 (5)	0.0472 (5)	0.0242 (4)	−0.0145 (4)	0.0123 (3)	−0.0023 (3)
O1	0.0936 (18)	0.0620 (14)	0.0352 (12)	−0.0157 (12)	0.0352 (12)	−0.0120 (10)
O2	0.0458 (13)	0.0743 (15)	0.0405 (12)	−0.0237 (11)	0.0049 (10)	0.0042 (10)
N1	0.0410 (13)	0.0363 (13)	0.0267 (12)	0.0040 (10)	0.0141 (10)	0.0016 (9)
N2	0.0485 (15)	0.0392 (13)	0.0404 (14)	−0.0012 (11)	0.0256 (12)	−0.0044 (10)
C1	0.0460 (18)	0.0491 (17)	0.0261 (14)	−0.0061 (14)	0.0077 (13)	0.0049 (12)
C2	0.057 (2)	0.055 (2)	0.062 (2)	−0.0071 (16)	0.0288 (18)	0.0050 (16)
C3	0.081 (3)	0.062 (3)	0.097 (3)	−0.021 (2)	0.042 (3)	0.011 (2)
C4	0.100 (3)	0.049 (2)	0.086 (3)	−0.008 (2)	0.035 (3)	0.011 (2)
C5	0.076 (3)	0.057 (2)	0.085 (3)	0.017 (2)	0.033 (2)	0.0152 (19)
C6	0.052 (2)	0.063 (2)	0.055 (2)	0.0026 (17)	0.0188 (17)	0.0143 (16)
C7	0.0410 (16)	0.0315 (14)	0.0298 (15)	−0.0055 (12)	0.0176 (13)	−0.0046 (11)
C8	0.0488 (18)	0.0485 (17)	0.0273 (15)	0.0014 (14)	0.0098 (14)	0.0058 (12)
C9	0.0415 (17)	0.0509 (18)	0.0411 (17)	0.0036 (14)	0.0128 (14)	0.0055 (14)
C10	0.055 (2)	0.060 (2)	0.0399 (17)	0.0137 (16)	0.0243 (15)	0.0035 (14)
O3	0.0665 (15)	0.0514 (13)	0.0562 (14)	−0.0079 (10)	0.0388 (12)	−0.0118 (10)

### Geometric parameters (Å, º)

**Table:**

P1—O2	1.477 (2)	C3—H3	0.9300
P1—O1	1.486 (2)	C4—C5	1.358 (5)
P1—C1	1.795 (3)	C4—H4	0.9300
P1—C7	1.830 (3)	C5—C6	1.382 (5)
N1—C7	1.348 (3)	C5—H5	0.9300
N1—C8	1.371 (3)	C6—H6	0.9300
N1—C10	1.455 (3)	C8—C9	1.341 (4)
N2—C7	1.329 (3)	C8—H8	0.9300
N2—C9	1.359 (3)	C9—H9	0.9300
N2—H20	0.8730	C10—H10A	0.9600
C1—C2	1.389 (4)	C10—H10B	0.9600
C1—C6	1.393 (4)	C10—H10C	0.9600
C2—C3	1.376 (4)	O3—H3A	0.8502
C2—H2	0.9300	O3—H3B	0.8510
C3—C4	1.369 (5)		
			
O2—P1—O1	122.39 (13)	C3—C4—H4	119.8
O2—P1—C1	109.20 (14)	C4—C5—C6	120.1 (4)
O1—P1—C1	109.76 (13)	C4—C5—H5	120.0
O2—P1—C7	107.60 (12)	C6—C5—H5	120.0
O1—P1—C7	103.61 (13)	C5—C6—C1	120.6 (3)
C1—P1—C7	102.25 (12)	C5—C6—H6	119.7
C7—N1—C8	109.3 (2)	C1—C6—H6	119.7
C7—N1—C10	126.2 (2)	N2—C7—N1	106.4 (2)
C8—N1—C10	124.5 (2)	N2—C7—P1	124.5 (2)
C7—N2—C9	110.1 (2)	N1—C7—P1	129.0 (2)
C7—N2—H20	122.7	C9—C8—N1	106.8 (3)
C9—N2—H20	127.0	C9—C8—H8	126.6
C2—C1—C6	118.2 (3)	N1—C8—H8	126.6
C2—C1—P1	120.6 (2)	C8—C9—N2	107.4 (2)
C6—C1—P1	121.3 (2)	C8—C9—H9	126.3
C3—C2—C1	120.5 (3)	N2—C9—H9	126.3
C3—C2—H2	119.8	N1—C10—H10A	109.5
C1—C2—H2	119.8	N1—C10—H10B	109.5
C4—C3—C2	120.3 (4)	H10A—C10—H10B	109.5
C4—C3—H3	119.8	N1—C10—H10C	109.5
C2—C3—H3	119.8	H10A—C10—H10C	109.5
C5—C4—C3	120.4 (4)	H10B—C10—H10C	109.5
C5—C4—H4	119.8	H3A—O3—H3B	108.5
			
O2—P1—C1—C2	−166.5 (2)	C9—N2—C7—P1	177.64 (19)
O1—P1—C1—C2	−29.7 (3)	C8—N1—C7—N2	0.5 (3)
C7—P1—C1—C2	79.8 (3)	C10—N1—C7—N2	−178.4 (2)
O2—P1—C1—C6	14.8 (3)	C8—N1—C7—P1	−177.3 (2)
O1—P1—C1—C6	151.5 (2)	C10—N1—C7—P1	3.7 (4)
C7—P1—C1—C6	−99.0 (2)	O2—P1—C7—N2	142.8 (2)
C6—C1—C2—C3	−0.2 (5)	O1—P1—C7—N2	11.9 (3)
P1—C1—C2—C3	−179.0 (3)	C1—P1—C7—N2	−102.2 (2)
C1—C2—C3—C4	0.2 (6)	O2—P1—C7—N1	−39.7 (3)
C2—C3—C4—C5	0.3 (6)	O1—P1—C7—N1	−170.6 (2)
C3—C4—C5—C6	−0.6 (6)	C1—P1—C7—N1	75.3 (3)
C4—C5—C6—C1	0.5 (5)	C7—N1—C8—C9	−0.5 (3)
C2—C1—C6—C5	−0.1 (4)	C10—N1—C8—C9	178.5 (3)
P1—C1—C6—C5	178.7 (3)	N1—C8—C9—N2	0.3 (3)
C9—N2—C7—N1	−0.3 (3)	C7—N2—C9—C8	0.0 (3)

### Hydrogen-bond geometry (Å, º)

**Table:**

*D*—H···*A*	*D*—H	H···*A*	*D*···*A*	*D*—H···*A*
O3—H3*A*···O1^i^	0.85	1.86	2.709 (3)	174
C10—H10*B*···O1^ii^	0.96	2.51	3.268 (4)	136
N2—H20···O3^iii^	0.87	1.82	2.665 (3)	162

Symmetry codes: (i) −*x*+1, −*y*, −*z*; (ii) *x*, *y*, *z*+1; (iii) *x*+1, *y*, *z*.
